# A framework for combining morbidity and mortality to identify determinants of child health

**DOI:** 10.3389/fpubh.2026.1709618

**Published:** 2026-07-15

**Authors:** Anuradha R. Chetiya, Vishal Deo

**Affiliations:** 1 Department of Statistics, Ramjas College, University of Delhi, New Delhi, India; 2 National Institute for Research in Digital Health, Indian Council of Medical Research, New Delhi, India

**Keywords:** child health, malnutrition, infant and child mortality, socioeconomic determinants, cumulative link mixed model, maternal factors, NFHS-5

## Abstract

**Objective:**

The objective of this study is to combine morbidity in terms of various levels of growth faltering or malnutrition among surviving children with mortality to better understand the determinants of infant and child health.

**Method:**

This study uses data from the latest National Family Health Survey (NFHS)-5 of India conducted during 2019–21 to provide a framework that integrates both mortality and morbidity as a measure of infant and child health to investigate their association with maternal and other socio-economic determinants of health. The outcome variable representing health status of a child is defined as a categorical variable with four progressively worsening levels– no malnutrition, moderate malnutrition, severe malnutrition, and not alive. The levels of morbidity in terms of growth faltering or malnutrition are defined in line with the World Health Organization (WHO) guidelines. Cumulative Link Mixed Model (CLMM) with partial proportional odds framework has been used to assess the association of risk factors with the outcome variable. Multivariate Imputations by Chained Equation has been employed to address high amount of missingness in three risk factors. Parameter estimates from CLMMs fitted to each of the 20 multiply imputed datasets were combined using Rubin’s rules to obtain pooled estimates.

**Results:**

Among the examined determinants, maternal education and household wealth index demonstrated the strongest associations with child health outcomes. Notably, low birth weight and non-institutional delivery are consistently strong risk factors across all thresholds of child health status, from moderate malnutrition till mortality. Antenatal care incompleteness does not associate with whether a child enters moderate malnutrition, but it strongly associates (14–19% higher odds) with severe malnutrition and with death, confirming its role in preventing the worse child health outcomes.

**Conclusion:**

Combining child mortality and morbidity allows better indication of child health status with added sensitivity through progressive health status levels, ranging from best scenario (no malnutrition) to worst scenario (death). Consequently, it enables a more comprehensive assessment of maternal and socioeconomic determinants of child health. In addition, using such combined outcome ensures complete utilization of information available in a survey dataset, irrespective of the mortality status of the child.

## 1 Introduction

Poor health in early childhood, including malnutrition or infections, can lead to chronic diseases in adulthood affecting the quality of life of an individual. Research studies have linked health of children in their early developmental age to non-communicable risk factors at the later stage of their life (1–6). Early childhood represents a window of opportunity for interventions like improving nutrition, vaccination, and healthcare access to ensure a healthier life. Investing in early childhood health lowers the economic burden on a country’s healthcare systems by preventing chronic illnesses that are often expensive to manage later in adulthood.

Research studies on infant and child health have focused on identifying determinants of the standard mortality rates like IMR (infant mortality rate), under five mortality rate and similar measures (2, 7–13). While mortality is the number of deaths and is absolute, morbidity is a measure of those affected by the health condition and is not absolute as there are chances that some may survive while others may not, or some may survive but with lower quality of health. So, it becomes pertinent that child health status be examined not just in the terms of mortality, but also by incorporating child growth parameters as outcomes of interest, which can together present a more comprehensive picture. The Mosley Framework (14) is a seminal analytical model designed to study child health, mortality, and morbidity. Developed by Mosley and Chen (14), it integrates social, economic, and biological factors to identify determinants of child health outcomes. The analytical framework proposed by Mosley and Chen (14) for child survival provides a basis for understanding the many factors that influence bringing up of a healthy child in a developing economy. While social scientists tend to focus on mortality, most medical scientists typically tend to focus on health status of survivors of a disease. Mosley and Chen (14) argued that focusing merely on mortality ‘handicaps research as death is a rare event’. They suggested integrating both approaches by combining counts of the dead with observations on the living to obtain a unified scale or index for determining the health status of a population. Based on the premise that, “in an optimal setting over 97 percent of newborns can be expected to survive through the first 5 years of life”, their framework provides a structured approach to understanding how interventions, such as improved healthcare, nutrition, or sanitation, can reduce child mortality and morbidity.

The present study draws motivation from the idea given by the Mosley-Chen framework. It combines morbidity in terms of various levels of growth faltering or malnutrition among surviving children, with mortality to better understand the determinants of infant and child health. This study uses data from the latest National Family Health Survey (NFHS)-5 (15) of India conducted during 2019–21, to provide a framework that incorporates both mortality and morbidity as a measure of infant and child health to investigate their relation to maternal and other socioeconomic determinants of health. While using demographic health survey data, like NFHS, taking mortality status of children as the outcome measure leads to non-utilization of information on children who may be alive but not healthy. In addition, the presence of censoring with respect to mortality outcome adds to the limitation of such analysis. The NFHS 5 provides data of children up to 5 years of age. So, for example, if the event of interest is under-five mortality, information on a child who is alive and less than 5 years of age at the time of the survey is censored as the event may occur in the future. Our framework overcomes both these limitations by incorporating health status of all children irrespective of their mortality status.

## 2 Methodology

### 2.1 Data and sample design

This analysis utilizes data from the fifth round of the NFHS conducted during 2019–21 in India. The NFHS is a nationally representative cross-sectional survey that collects data on a large number of demographic, socioeconomic, maternal and child health parameters. The previous four rounds of the NFHS were conducted in 1992–1993 (NFHS-1), 1998–1999 (NFHS-2), 2005–2006 (NFHS-3) and 2015–2016 (NFHS-4). The NFHS surveys were carried out under the guidance of the Ministry of Health and Family Welfare (MoHFW), Government of India with the International Institute for Population Sciences (IIPS), Mumbai, as the nodal agency. The overall sample size for NFHS-5 was based on several factors, the most important being the requirement to generate indicators at the district and/or state and union territory (UT) levels. NFHS-5 was conducted in 707 districts, 28 states, and 8 union territories. In this round of NFHS 5, a two-stage stratified sampling approach has been used. Each district was divided into urban and rural strata. From each rural sampling stratum, villages were selected as Primary Sampling Units (PSUs). For the urban strata, Census Enumeration Blocks (CEBs) were selected as PSUs. A total of 664,972 households were selected for the sample, out of which 653,144 were found to be occupied. Interviews were successfully conducted in 636,699 of these households, yielding a response rate of 98 percent. Within the interviewed households, 747,176 women aged 15–49 were identified as eligible for individual interviews, and interviews were successfully completed with 724,115 women, resulting in a response rate of 97 percent (17). Data from the children recode file was used in this analysis.

### 2.2 Dependent variable

To incorporate both mortality and morbidity in the analysis, four levels of the dependent variable were defined. Three levels of morbidity in terms of growth faltering or malnutrition were defined in line with the World Health Organization (WHO) guidelines. The WHO classification for malnutrition considers standard deviation or Z scores of weight for length/height for classifying children in moderate (between −2 and −3 SD) and severe (<−3SD) categories (16). For our analysis the four levels of the dependent variable that we have considered are, 1: No Malnutrition (greater than −2SD), 2: Moderate Malnutrition (between −2 to −3SD), 3: Severe Malnutrition (less than −3SD), and 4: Not Alive. That is, the dependent variable level 1 represents the best possible outcome and level 4 represents the worst possible outcome.

### 2.3 Independent variables

Socioeconomic and demographic factors identified in various studies include nutritional status of mother, age of the mother, gaps between two deliveries, mother’s education level, access to healthcare services that ensure safe delivery along with antenatal and postnatal care (2, 8–12, 16). Since maternal health is an immediate and principal factor in determining child mortality, we classified one category of independent variables under the umbrella of maternal factors and the second category of independent variables under environmental factors. This classification follows the broader framework of socioeconomic determinants that influence risk of mortality and morbidity in a developing country given by Mosley and Chen (14). Data on all variables included as predictor variables in the study are available in the child recode file.

#### 2.3.1 Maternal variables

The following maternal variables were considered for analysis.

*Age of the mother at the time of birth*: For our analysis we have classified current age of mother under three categories −15–24 years, 25–34 years, and 35–49 years using data on mother’s age from the child recode file.

*Birth order*: Birth order of the child considered in the study was classified into three groups viz. first, second or third, fourth or more.

*Full AnteNatalCare (ANC) Utilization:* A mother is considered to have received full ANC (ante natal care) if she received minimum required TT dosages, consumed prophylactic IFA supplement for at least 100 days, and completed four or more ANC visits is considered to have received full ANC.

*Place of delivery*: The NFHS compiles data on the place of delivery across 17 categories, encompassing delivery at the respondent’s home, another home, a parent’s home, hospitals, public health facilities, primary health centers, dispensaries, clinics, as well as both private and public healthcare facilities. All deliveries other than respondent’s home, other home and parent’s home have been considered as institutional deliveries. And all home deliveries have been taken as non-institutional deliveries.

*Educational level of the mother*: In India, the school education system comprises 10 + 2 years of schooling, including 10 years of primary and secondary education followed by 2 years of higher secondary education. For this variable, four categories of education level were defined – mothers with no education, mothers who have completed primary education, secondary education and higher education, respectively.

*Birth weight*: A child under 2,500 grams has been considered as underweight and all children above this weight has been considered as having normal birth weight. This is according to the WHO definition of ‘low birth weight’.

#### 2.3.2 Environmental variables

The following variables have been included in the category of environmental variables for the analysis.

*Wealth index*: The NFHS-5 survey assessed the economic status of households using a measure called the wealth index. This index assigned scores to households based on the quantity and type of consumer goods they owned, such as a television, bicycle, car, or motorcycle, as well as housing characteristics like the source of drinking water, toilet facilities, and flooring type. Households were then categorized into five wealth quintiles, each representing 20% of the population. All five quintiles (lowest, second, middle, fourth, and highest) have been included in the analysis.

*Place of residence:* The types of place of residence have been taken as rural and urban households. A total 14,816 urban and 42,954 rural households have been included in the analysis.

*Cooking fuel:* NFHS data is available for more than 10 categories of cooking fuel used. For this analysis we have considered electricity, liquefied petroleum gas, natural gas and biogas as safe fuels and the remaining fuel sources which includes kerosene, charcoal, wood, animal dung and agricultural crop among others as unsafe fuels.

*Sex of the child*: Whether the child is male or female are the two categorizations for this variable. The environment significantly influences gender development in children specially in developing economies. Social, cultural, and economic factors often shape gender roles and expectations from an early age. Issues like poverty, urban crowding, and lack of infrastructure can exacerbate gender inequalities particularly affecting girls thereby impacting their growth and mental health.

### 2.4 Statistical analysis

#### 2.4.1 Missing data and preliminary analysis

**Figure 1:**
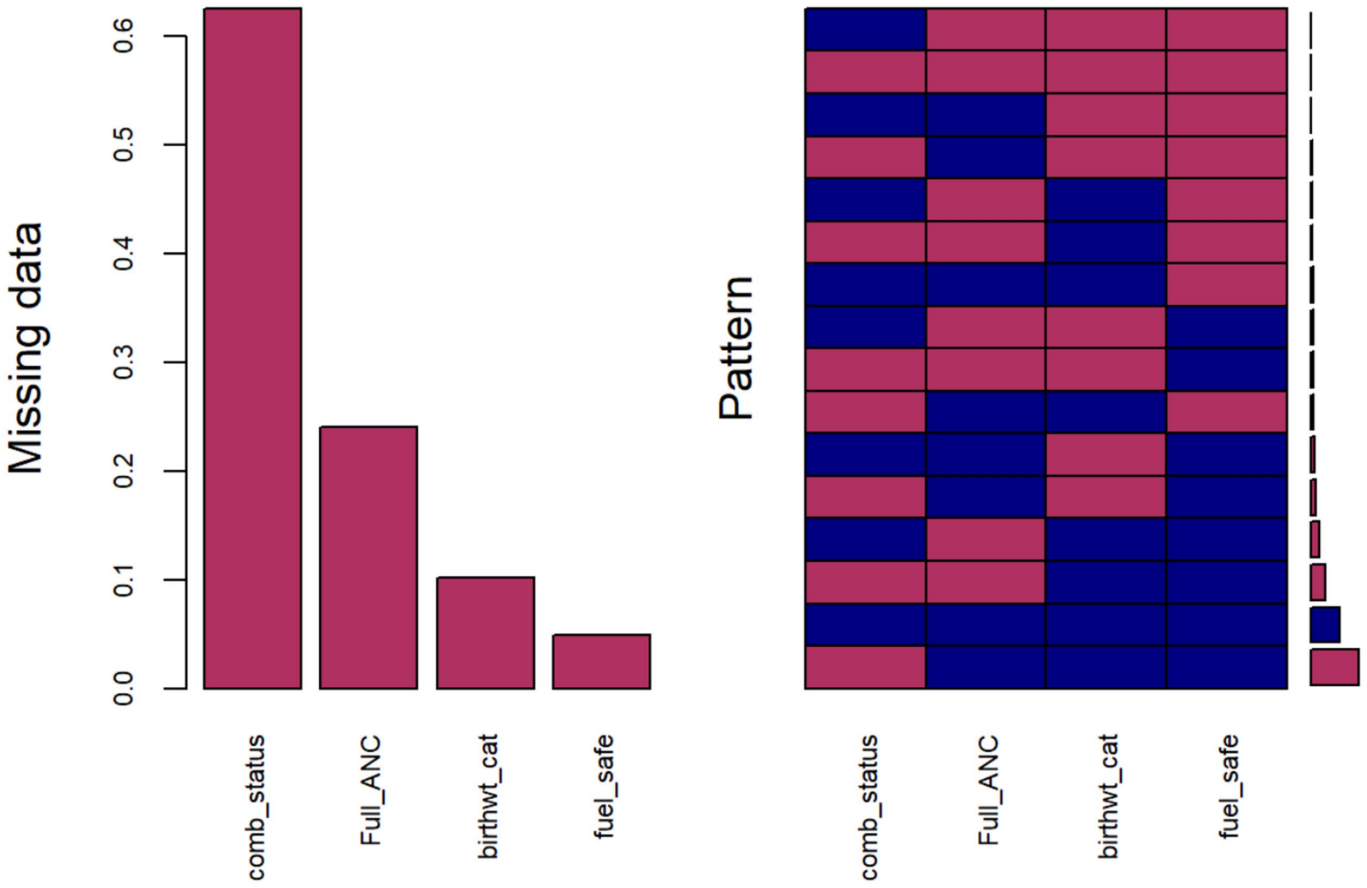
Scale and pattern of missingness in the factor variables.

The total number of children under 5 years of age in the child recode file were 232,920. Out of these, complete information on the variables of interest was available for only 57,770 children. Missing values were present in three of the factor variables considered in the study- *full ANC status, birth weight category, and cooking fuel*. Among these three factors, maximum number of missingness was found in the variable *full ANC status* (21,148). In addition, the derived outcome variable *comb_status* had a significant amount of missingness (145,656). A summary of scale and pattern of missingness is presented in the Figure 1. To assess the nature of missingness, the proportion of missing values was examined across subgroups of each covariate. Patterned missingness concentrated within specific categories would suggest departure from the Missing Completely at Random (MCAR) assumption and potential bias in complete-case analysis. For example, the pattern of missingness in full ANC status at different levels of other factors is provided in Table 1.

**Table 1:** Missingness proportion in the variable Full_ANC at different levels of the other factor variables.

Type of place of residence	
Urban	20%
Rural	26%
Wealth index combined	
Poorest	31%
Poorer	26%
Middle	23%
Richer	21%
Richest	17%
Highest education level	
No education	30%
Primary	28%
Secondary	23%
Higher	17%
Sex of child	
Male	22%
Female	27%
Birth weight category	
Low birth weight	26%
Normal weight	22%
Place of delivery	
Non-institutional	34%
Institutional	23%
Cooking fuel	
Safe	20%
Unsafe	28%
Birth order number	
First	34%
Second or third	18%
Fourth or more	19%
Mother’ age	
15–24	26%
25–34	25%
35–49	16%

To get a preliminary understanding of bivariate association between each factor and the outcome variable, Chi-square test for independence of attributes was used on the complete cases. Survey weights, provided as the variable v005 in the individual records file of the NFHS-5 dataset, were used to obtain weighted proportions and design-adjusted chi-square test statistics. v005 is a probability-based sampling weight assigned to each woman respondent (aged 15–49 years) to correct for the unequal probability of selection arising from the complex, multi-stage stratified cluster design of NFHS-5. It ensures that weighted estimates are nationally and sub-nationally representative. A summary of the maternal and environmental factors and their bivariate association with the dependent variable is provided in Table 2.

**Table 2:** Bivariate associations of maternal and environmental factors with the dependent variable on complete cases.

	Health status category					
Factors	No malnutrition	Moderate malnutrition	Severe malnutrition	Not alive	Total	Test
	34,637	17,674	2,340	3,119	57,770	
Place of delivery						
Non-institutional	1,166 (3.2%)	1,452 (8.3%)	215 (8.9%)	250 (8.1%)	3,083 (5.2%)	<0.001
Institutional	35,397 (96.8%)	16,014 (91.7%)	2,207 (91.1%)	2,832 (91.9%)	56,450 (94.8%)	
Cooking fuel safe/unsafe						
Safe	25,812 (70.6%)	6,832 (39.1%)	999 (41.3%)	1,340 (43.5%)	34,983 (58.8%)	<0.001
Unsafe	10,751 (29.4%)	10,635 (60.9%)	1,422 (58.7%)	1,742 (56.5%)	24,550 (41.2%)	
Birth order						
First	13,329 (36.5%)	5,220 (29.9%)	622 (25.7%)	973 (31.6%)	20,144 (33.8%)	<0.001
Second or third	20,471 (56.0%)	9,191 (52.6%)	1,315 (54.3%)	1,451 (47.1%)	32,428 (54.5%)	
Fourth or more	2,762 (7.6%)	3,056 (17.5%)	484 (20.0%)	658 (21.4%)	6,961 (11.7%)	
Mother’s current age						
15–24	10,315 (28.2%)	4,949 (28.3%)	422 (17.4%)	1,086 (35.2%)	16,772 (28.2%)	<0.001
25–34	22,839 (62.5%)	10,574 (60.5%)	1,568 (64.7%)	1,620 (52.6%)	36,600 (61.5%)	
35–49	3,409 (9.3%)	1,943 (11.1%)	432 (17.8%)	376 (12.2%)	6,160 (10.3%)	
Type of place of residence						
Urban	15,197 (41.6%)	3,216 (18.4%)	438 (18.1%)	721 (23.4%)	19,572 (32.9%)	<0.001
Rural	21,365 (58.4%)	14,250 (81.6%)	1,984 (81.9%)	2,361 (76.6%)	39,961 (67.1%)	
Wealth index combined						
Poorest	2,921 (8.0%)	6,084 (34.8%)	874 (36.1%)	888 (28.8%)	10,767 (18.1%)	<0.001
Poorer	5,272 (14.4%)	4,471 (25.6%)	585 (24.2%)	800 (25.9%)	11,127 (18.7%)	
Middle	7,401 (20.2%)	3,209 (18.4%)	416 (17.2%)	565 (18.3%)	11,592 (19.5%)	
Richer	9,714 (26.6%)	2,389 (13.7%)	343 (14.2%)	561 (18.2%)	13,008 (21.9%)	
Richest	11,254 (30.8%)	1,314 (7.5%)	203 (8.4%)	268 (8.7%)	13,039 (21.9%)	
Highest educational level of mother						
No education	3,282 (9.0%)	4,724 (27.0%)	749 (30.9%)	874 (28.4%)	9,629 (16.2%)	<0.001
Primary	3,060 (8.4%)	2,457 (14.1%)	319 (13.2%)	453 (14.7%)	6,289 (10.6%)	
Secondary	20,294 (55.5%)	8,611 (49.3%)	1,104 (45.6%)	1,423 (46.2%)	31,432 (52.8%)	
Higher	9,927 (27.1%)	1,675 (9.6%)	250 (10.3%)	332 (10.8%)	12,184 (20.5%)	
Sex of child						
Male	19,754 (54.0%)	9,602 (55.0%)	1,293 (53.4%)	1,709 (55.5%)	32,359 (54.4%)	0.343
Female	16,809 (46.0%)	7,864 (45.0%)	1,128 (46.6%)	1,373 (44.5%)	27,174 (45.6%)	
Full ANC status						
No	21,782 (59.6%)	12,429 (71.2%)	1,711 (70.6%)	2,296 (74.5%)	38,218 (64.2%)	<0.001
Yes	14,781 (40.4%)	5,037 (28.8%)	711 (29.4%)	786 (25.5%)	21,315 (35.8%)	
Birth weight category						
Low birth weight	5,454 (14.9%)	3,800 (21.8%)	576 (23.8%)	1,065 (34.6%)	10,895 (18.3%)	<0.001
Normal weight	31,108 (85.1%)	13,667 (78.2%)	1,846 (76.2%)	2,017 (65.4%)	48,638 (81.7%)	

#### 2.4.2 Statistical model

Since the proposed dependent variable has ordinal scale, multivariable ordinal logistic regression models were initially considered. Ordinal logistic regression assumes proportional odds, meaning that the effect of each predictor is assumed to be constant across all cumulative thresholds of the ordered dependent variable. Wolfe–Gould, Brant, and likelihood ratio tests indicated that this proportional odds assumption was statistically significantly violated for all factors except for the factor *Child’s sex at birth*. Consequently, a Cumulative Link Mixed Model (CLMM) was fitted to examine the determinants of child health outcome defined as an ordered four-level variable ranging from “No Malnutrition” (best outcome) through “Moderate Malnutrition” and “Severe Malnutrition” to “Not Alive” (worst outcome). Since the proportional odds assumption was violated for most factors, the model was estimated under the partial proportional odds framework: child’s sex at birth was modelled under the proportional hazards assumption (yielding a single common odds ratio across all transitions), while all remaining predictors were treated as nominal factors, producing transition-specific odds ratios (ORs) for three cumulative thresholds: No Malnutrition vs. Moderate/Severe/Not Alive (Threshold 1), Moderate vs. Severe/Not Alive (Threshold 2), and Severe Malnutrition vs. Not Alive (Threshold 3). Models were fitted using the *clmm2*() function available in the R package *ordinal* (18). The models were fitted while accounting for survey weights, i.e., the probability-based sampling weight assigned to each woman respondent (aged 15–49 years). Survey design with weights defined by the respondent-level probability-based sampling weight and clusters defined by State/Union Territory (UT) was constructed using the R package *survey* (19).

In view of the hierarchical clustered nature of the survey data, multilevel model has been fitted with States/Union Territories (UTs) acting as the second level. In the process of identifying best model, four models were fitted progressively. The first model was fitted with intercept and the factors *Child’s sex at birth* and *Birth weight,* and served as the base model. Subsequently, *Birth order*, *Mother’s current age*, and *Highest educational level of mother* were added to the base model to obtain the second model. *Full ANC status* and *Place of delivery* were further added as factors to obtain the third model. In the final model, all remaining factors, viz., *Cooking fuel*, *Type of place of residence*, and *Wealth index combined* were also included. All models were fitted with random effect for the State/UT variable to account for the multi-level heterogeneity. Models were compared based on their Akaike Information Criterion (AIC) and Bayesian Inference Criterion (BIC) values. The model which accommodated all factors of interest of the study was found the best with lowest AIC and BIC values, and it has been used for the final analysis presented in the results section.

#### 2.4.3 Multiple imputation

**Figure 2:**
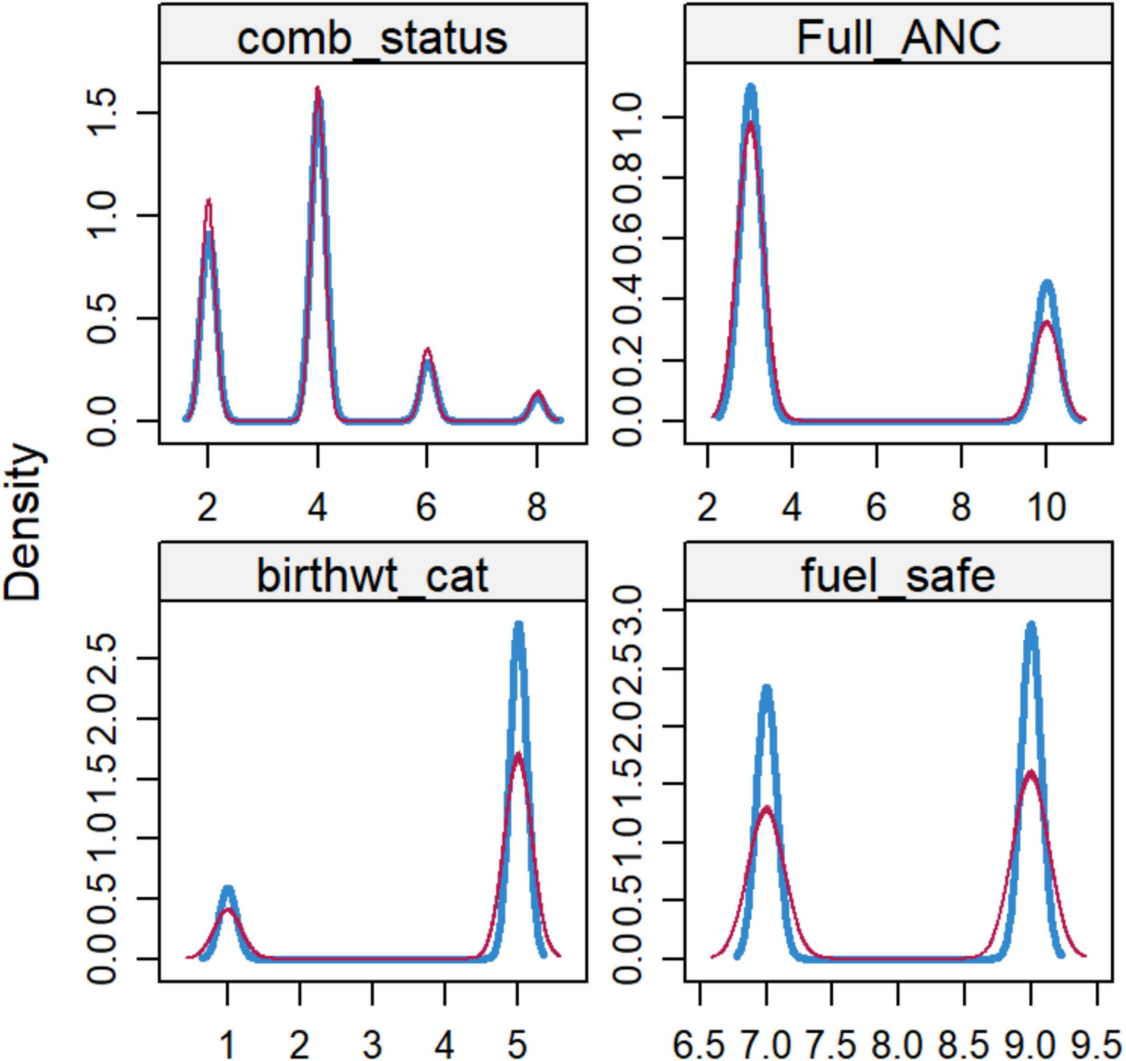
Kernel densities of the observed and imputed values of the four variables with missingness. The blue line is the kernel density of the observed (non-missing) values, and the red line is the pooled density of the imputed values across all 20 imputed datasets.

Scale and pattern of missingness, as apparent from Figure 1 and Table 1, suggest that analysis of only complete cases may induce bias in the results. Since there is a huge amount of missingness in the data, with only 25% of the data being complete, and also, since some patterns in missingness was seen across some specific categories, the CLMM model was fitted on 20 multiple-imputed datasets. Multiple imputation was carried out using the Multivariate Imputations by Chained Equation (MICE) method and was executed in R using the package *mice* (20, 21). Imputations were done for missing values in the three factor variables and the outcome variable, and all other non-missing factor variables and design variables (cluster variable and weights) were also included as covariates. Convergence of the imputation models was assessed using convergence plots function in the *mice* package. To assess the plausibility of the imputed data, kernel densities of the observed and pooled imputed data of the four variables were plotted using the *densityplot*() function the R package *mice* (Figure 2). Since the outcome variable was also included in the multiple imputation, Multiple Imputation, then Deletion (MID) approach was followed, where the cases with imputed values of the outcome variable were not included in the fitting of the model (22). Consequently, CLMM was fitted on the imputed dataset of size 87,264 individuals. Pooled estimates of regression coefficients (and odds ratios), standard errors, and confidence intervals were obtained using Rubin’s rule (20).

**Figure 3:**
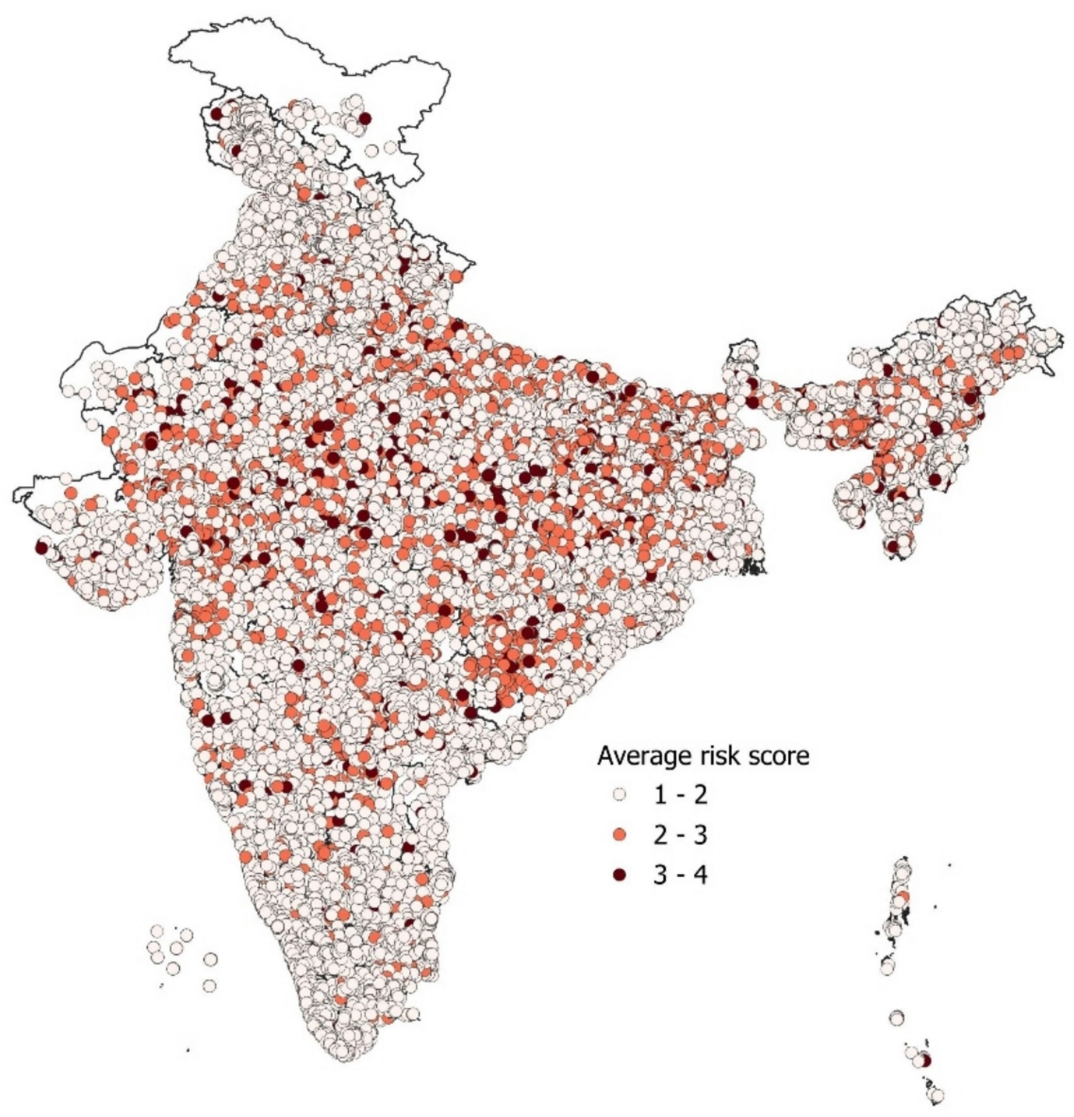
Map showing average risk scores across clusters defined in NFHS-5.

Weighted average values of the dependent variable, with weights defined by respondent-level survey weights, have been plotted as a choropleth graph in Figure 3 for the clusters defined in the NFHS 5 sampling design (15) to present a spatial distribution of the high-risk zones. The map was plotted using the software QGIS version 3.40.9.

## 3 Results

In Figure 2, each panel represents one variable with missing data. The blue line is the kernel density of the observed (non-missing) values, and the red line is the pooled density of the imputed values across all 20 imputed datasets. Good imputations are expected to produce a distribution similar to the observed data. However, an exact match is not always expected or required. For the outcome variable *comb_status*, and the factor *Full_ANC*, the red and the blue lines almost overlap. Although there is a slight discrepancy at the peaks in case of the variables *birthwt_cat* and *fuel_safe*, there is no systematic shift between blue and red lines, and the result can be deemed acceptable.

Bivariate analysis on the complete cases revealed that all examined factors were significantly associated with the outcome variable, except for the sex of child. However, these associations were unadjusted and do not account for confounding with other variables. Therefore, to better understand the marginal effect of each factor, results from the CLMM estimated under partial proportional odds framework will be interpreted. Pooled result of the CLMM fitted on the 20 imputed datasets is provided in Table 3. The estimated random-effect variance was 0.414 (95% CI: 0.237–0.713), confirming significant between-cluster heterogeneity and justifying the use of a mixed-effects specification. It indicates that there are significant, systematic differences in the average outcome (intercept) across different States/UTs and that the multilevel model is well suited for the analysis.

**Table 3:** Pooled result (Rubin’s rule) of the CLMMs fitted on 20 multiple imputation datasets (*n* = 87,264).

*Factors*	Threshold 1: No Malnutrition|Moderate malnutrition			Threshold 2: Moderate Malnutrition|Severe malnutrition			Threshold 3: Severe Malnutrition|Not alive		
	OR	95% CI	*p*-value	OR	95% CI	*p*-value	OR	95% CI	*p*-value
*Child’s sex at birth (b4)–Base category: male*									
Female	0.93	[0.90–0.95]	<0.000	0.93	[0.90–0.95]	<0.000	0.93	[0.90–0.95]	<0.000
*Birth weight–Base category: Normal birth weight*									
Low birth weight	0.59	[0.56–0.61]	<0.000	0.47	[0.45–0.50]	<0.000	0.43	[0.40–0.45]	<0.000
*Birth order–Base category: First*									
Second or third	1.25	[1.21–1.30]	<0.000	1.21	[1.15–1.27]	<0.000	1.22	[1.16–1.29]	<0.000
Fourth or more	1.21	[1.14–1.28]	<0.001	1.06	[0.98–1.13]	0.135	0.99	[0.91–1.07]	0.762
*Mother’s current age (baseline)–Base category: 15–24*									
25–34	0.76	[0.73–0.79]	<0.000	0.96	[0.91–1.01]	0.093	1.24	[1.18–1.31]	<0.000
35–49	0.65	[0.61–0.69]	<0.000	0.79	[0.73–0.85]	<0.000	1.33	[1.2–1.46]	<0.000
*Highest educational level of mother (v106)–Base category: No education*									
Primary education	1.27	[1.20–1.34]	<0.000	1.11	[1.04–1.18]	0.002	1.02	[0.95–1.10]	0.531
Secondary education	1.46	[1.39–1.53]	<0.000	1.27	[1.21–1.34]	<0.000	1.27	[1.20–1.35]	<0.000
Higher education	2.11	[1.98–2.24]	<0.000	1.56	[1.43–1.70]	<0.000	1.64	[1.49–1.81]	<0.000
*Full ANC status–Base category: Yes*									
No	0.98	[0.93–1.03]	0.471	0.86	[0.81–0.92]	<0.000	0.79	[0.73–0.86]	<0.000
*Place of delivery–Base category: Institutional*									
Non-Institutional	0.75	[0.71–0.79]	<0.000	0.80	[0.75–0.84]	<0.000	0.77	[0.72–0.82]	<0.000
*Cooking Fuel–Base category: Safe*									
Unsafe	0.83	[0.80–0.87]	<0.000	0.93	[0.88–0.98]	0.008	0.89	[0.84–0.95]	<0.000
*Type of place of residence (V025)–Base category: urban*									
rural	0.79	[0.76–0.82]	<0.000	0.91	[0.86–0.96]	0.001	0.96	[0.90–1.02]	0.171
*Wealth index combined (v190): Base category: Poorest*									
Poorer	2.02	[1.93–2.12]	<0.000	1.15	[1.08–1.21]	<0.000	1.04	[0.97–1.11]	0.242
Middle	3.17	[3.00–3.34]	<0.000	1.39	[1.30–1.49]	<0.000	1.21	[1.12–1.30]	<0.000
Richer	4.70	[4.42–4.99]	<0.000	1.70	[1.57–1.84]	<0.000	1.40	[1.08–1.42]	<0.000
Richest	8.73	[8.10–9.40]	<0.000	2.77	[2.50–3.08]	<0.000	2.25	[2.01–2.53]	<0.000
*Intercept*	0.71	[0.56–0.90]	0.005	8.15	[6.36–10.44]	<0.000	12.37	[9.59–15.96]	<0.000
*Estimate of random effect variance [95% CI]*						0.414 [0.237–0.713]			

OR, Odds Ratio.

While the variable full ANC status accounts for maximum number of missingness among the factor variables (Figure 1), from Table 1 we can also see that missingness in the variable is higher for children belonging to certain categories. These categories include children residing in rural regions, belonging to poorest or poorer households, with mothers having no or only primary education, who are female born, who are born at a non-institutional place, who are in household using unsafe cooking fuel, who are first born, and who are born to a mother of age 15–24. In addition, all these categories are historically considered as possibly associated with higher risk of worse health outcomes of children. This indicates that the model fitted on the complete cases only may lead to biased results. Consequently, while we did compare the results obtained from the models fitted on complete cases and on imputed datasets, the final interpretation of the association of the factors with the outcome is based on the pooled result of the imputation-based models provided in Table 3. While comparing the results from the two approaches, it was observed that most of the estimates remained consistent in their interpretation and effect size across both models. In some cases, estimates which were insignificant in the complete case analysis became statistically significant on using the imputed datasets, however, the direction of association remained consistent. Result of the CLMM fitted on the complete cases only is provided in Appendix A, Table A1.

### 3.1 Evidence from the CLMM fitted under the partial proportional odds framework

For the nominal factors, transition-specific odds ratios (ORs) for three cumulative thresholds are provided in Table 3: No Malnutrition versus (vs.) Moderate/Severe/Not Alive (Threshold 1), Moderate vs. Severe/Not Alive (Threshold 2), and Severe Malnutrition vs. Not Alive (Threshold 3). That is, the Threshold 1 (T1) discriminates well-nourished children from any level of malnutrition or death, Threshold 2 (T2) discriminates moderately malnourished from severely malnourished or death, and the Threshold 3 (T3) discriminates severely malnourished from child mortality. The results of this adjusted analysis based on imputed datasets provided the following insights into the associations of maternal and environmental factors with child health outcomes.

### 3.2 Maternal factors

#### 3.2.1 Mother’s age category (vs. 15–24)

Compared to the youngest mothers (aged 15–24), older maternal age groups demonstrated a complex, threshold-dependent pattern. At T1, children born to older mothers are associated with worse odds of avoiding malnutrition. However, at T3, older mothers’ children have higher odds of surviving severe malnutrition (OR > 1). This reversal suggests older mothers may be less able to prevent malnutrition onset but better equipped, possibly through experience, to prevent child death. This is a strong justification for the nominal treatment of this variable.

#### 3.2.2 Birth order (vs. first)

Children of second or third birth order had significantly higher odds of a better health outcome compared to first-born children, with ORs of 1.25 (95% CI: 1.21–1.30), 1.21 (95% CI: 1.15–1.27), and 1.22 (95% CI: 1.16–1.29) at T1, T2, and T3, respectively (all *p* < 0.001). That is, second/third-order children have consistently around 21–25% higher odds of better health status than first-born children across all transitions. Children of fourth or higher birth order showed a similar but diminished advantage at T1 (OR = 1.21, 95% CI: 1.14–1.28, p < 0.001); however, this advantage was not statistically significant at T2 (OR = 1.06, *p* = 0.135) or T3 (OR = 0.99, *p* = 0.762), suggesting that fourth or higher birth order protects against initial malnutrition entry but not against the most severe deteriorations.

#### 3.2.3 Full ANC (no full ANC vs. full ANC)

Not receiving full antenatal care also shows a threshold-varying pattern. Incomplete ANC has no significant effect at the T1 but becomes increasingly important at higher severity thresholds. This is a substantively meaningful finding suggesting ANC incompleteness does not associate with whether a child enters malnutrition, but it strongly associates (14–19% higher odds) with moderate or severe malnutrition and with death, confirming ANC’s role in preventing the worse child health outcomes.

#### 3.2.4 Mother’s education (vs no education)

Mother’s education shows a strong protective effect, with protection increasing progressively by the level of education. However, the protective effect declines at subsequent Threshold levels. The benefit of education is largest at preventing the onset of malnutrition (T1) and remains substantial at T2 and T3. Notably, primary education becomes non-significant at T3, meaning only secondary or higher education provides meaningful protection against child mortality once severe malnutrition has occurred. Higher education women have more than 2 times the odds of keeping their child well-nourished as compared to non-educated mothers, the strongest education effect in the model.

#### 3.2.5 Birth weight (normal vs. low)

Low birth weight is a strong, consistent risk factor at all thresholds. The effect strengthens as severity increases, with 41% lower odds of no malnutrition as compared to worse outcomes, 53% lower odds of being only moderately (not severely) malnourished, and 57% lower odds of being severely malnourished as compared to mortality. Low birth weight children are not only more likely to enter malnutrition, they are progressively more likely to deteriorate to severe malnutrition and mortality.

### 3.3 Environmental factors

Several environmental and socioeconomic factors were found to significantly influence child health outcomes.

#### 3.3.1 Residence (rural vs. urban)

Rural children are associated with higher odds of falling into worse combined health status categories, even after controlling for wealth, education, and other covariates. To be specific, rural residence reduces odds of better child health status significantly at T1 (21% lower odds) and T2 (9% lower odds), but not at T3.

#### 3.3.2 Cooking fuel (unsafe vs. safe)

Use of unsafe fuel at home is associated with worse child health outcomes at all thresholds. The effect is strongest at T1 (17% lower odds of avoiding any malnutrition), moderates at T2, then re-strengthens at T3. This suggests unsafe fuel has a broad impact on initial nutritional status and also contributes to the most severe outcomes.

#### 3.3.3 Place of delivery (non-institutional vs. institutional)

Non-institutional delivery is a persistent and uniform risk, reflecting poorer access to skilled birth attendance and postnatal care. Results show around 20–25% reduction in cumulative odds of better child health status at each threshold, with broadly similar magnitudes across all thresholds.

#### 3.3.4 Wealth index (vs poorest)

Household wealth is among the most powerful protective factor with a dramatic upward gradient in protection as wealth quintile increases. The wealth effect is most pronounced at T1 and declines markedly at higher thresholds. Richest quintile children have nearly 9 times the odds of being well-nourished vs. any malnutrition or death compared to the poorest, yet this drops to 2.77 times at T2, and 2.25 times at T3. Notably, the “Poorer” quintile is non-significant at T3 (OR = 1.04, *p* = 0.242), meaning at the critical severe-to-death threshold, being slightly less poor offers no survival advantage.

#### 3.3.5 Child’s sex (female vs. male) [proportional odds]

Since the Child’s sex factor variable met the proportional odds condition, Female sex has a single common OR = 0.93 (95% CI: 0.90–0.95, *p* < 0.001) applied uniformly across all thresholds. Female children have 7% lower cumulative odds of being in a better nutritional category compared to males, indicating a modest but consistent disadvantage for girls across all health status thresholds.

The choropleth map of weighted average risk scores (Figure 3) shows higher risks of child mortality and morbidity outcomes in India’s central and northern states revealing significant regional disparities in child health. These areas often exhibit higher rates of malnutrition, limited access to healthcare services, and poor sanitation, contributing to adverse child health outcomes. States like Uttar Pradesh, Bihar, Madhya Pradesh, Rajasthan, and Jharkhand have higher concentration of high-risk clusters. These states are part of the Empowered Action Group (EAG) states that were identified by the government of India for special attention due to their socio-economic and demographic challenges which included addressing issues of maternal and child mortality. In contrast, states in southern and western India exhibit lower risk scores.

## 4 Discussion

The categorical dependent variable used in this analysis provides a simple yet effective means of integrating information on under-five mortality and malnutrition to generate a comprehensive risk score for assessing child health status in a region. It enables the classification of child health status in a region along a continuum, from best (healthy) to worst (mortality), thereby offering deeper insights than traditional metrics such as under-five mortality, infant mortality, or malnutrition alone. For example, from the choropleth graph in Figure 1, various clusters within Rajasthan can be seen to have very high-risk scores. However, as per the NFHS 5 report, the state performs better than 12 States/UTs in terms of under-5 mortality (17).

Two most important determinants of child health outcome in terms of mortality and child growth factors that emerge from this study are- higher education among the maternal factors and wealth index among the environmental factors. The place of residence is significant with children in rural areas more likely to be in worse health status category as compared to those in urban areas, even after accounting for differences in wealth, education, and other factors. Both these results highlight the importance of higher education and poverty reduction especially in rural areas of the country. The gross enrollment rate (GER) in primary school in India is 100% indicating that almost all children of the eligible age group are enrolled in primary education (23). However, there is a significant drop to 57.6% at the higher secondary level (23). A large portion of students do not transition to or complete higher secondary education.

The global multidimensional poverty index (MPI) is a poverty measure that reflects the multiple deprivations that poor people face in areas of education, health, and living standards. As per the global multidimensional poverty report 2024, India is home to the largest population living in extreme poverty, totaling 234 million people (24). However, the report also highlights that India has demonstrated massive historic progress in poverty reduction, decreasing from 55.1% in 2005–2006 to 16.4% in the 2019–2021. A twin focus on education and poverty reduction can significantly improve child health outcomes by addressing the root causes of poor health. There is a need for stronger policies and interventions to improve retention, affordability, and accessibility of education at higher levels particularly in rural areas. Education empowers families with knowledge about nutrition, hygiene, and healthcare, thereby enabling them to make informed decisions for their children’s well-being. At the same time, creating jobs and reducing poverty provide families with the financial resources needed to access essential services like medical care, clean water, and nutritious food. For instance, educated parents are more likely to prioritize immunizations and seek timely medical interventions, reducing child mortality and morbidity. A stable family income ensures that children receive adequate nourishment and a healthy living environment, which is essential for their growth and well-being.

Full antenatal care (ANC) services emerged as a key protective factor against child mortality. Community-based awareness programs, including some digital health initiatives should prioritize educating women and their partners on the critical importance of timely and complete ANC utilization to maximize child health benefits. In addition, birth weight of child is a strong risk factor associated with child health status. It is interesting to note that, even after adjusting for maternal and environmental factors, female children have higher chance of being malnourished or experiencing mortality as compared to male children. Although the odds ratio of 7% indicate modest difference, it signifies further strengthening of policy interventions by the government that has been promoting equal healthcare access, nutritional interventions, maternal and child welfare measures, regardless of their gender.

## 5 Conclusion

India has made significant strides in improving child survival rates over the years. However, the country still registered unacceptably high child mortality rates as evident from the data of the latest NFHS 5. Among the G20 nations, a consortium of 19 countries and the European Union that together represent around 85% of the global GDP, over 75% of the global trade, and about two-thirds of the world population,^1^ India has the highest neonatal, infant, and under five mortality rates at 20.3, 27 and 32.6 per thousand live births, respectively.^2^ Given the diversity and the complex interaction of many factors there is no one size fit all solution to this problem. One needs to continuously examine data to draw relevant inferences for timely interventions. Combining child mortality and morbidity (in terms of malnutrition) to assess the child health outcome as proposed in this study allows better indication of child health status with added sensitivity through progressive health status levels, ranging from best scenario (no malnutrition) to worst scenario (death). Consequently, it enables a more comprehensive assessment of maternal and environmental determinants of child health. In addition, using such combined outcome ensures complete utilization of information available in a survey dataset, irrespective of the mortality status of the child. Identifying the determinants using this approach will not only identify factors that affect child mortality but also lead to a better understanding of the determinants of an improved quality of life with regard to the health of surviving children. For example, it is apparent from the result of the CLMM model that while some risk factors show consistent association with different thresholds of health outcome traversing malnourishment and mortality, some factors, such as place of residence (urban/rural), or incomplete ANC may be associated with certain specific thresholds only. Generating such insights may not be possible if we use only mortality or malnutrition as an outcome variable.

## 6 Limitations

This study proposes an alternative approach to defining and analyzing the health status of children. However, certain theoretical limitations exist which may be addressed in subsequent research. Firstly, in estimating the weighted average risk score for a cluster while plotting the Choropleth graph, the categories of the proposed outcome variable were treated with equal weights. That is, it is assumed that the categories are equally spaced, which may not be realistic. Future research may explore the assignment of differential weights to these categories to enable a more accurate and context-sensitive representation of regional risk scores. In addition, the imputation models (MICE) used in this study did not have any option for using a partial proportional hazards framework for the CLMM. Consequently, for imputing the outcome variable, ‘*ploreg*’ method was used which treats the categories as unordered. Development of imputation models for partial proportional odds CLMM may also be taken up as a research problem, which can further strengthen the use of such combined health status outcome variables.

## Data Availability

Publicly available datasets were analyzed in this study. This data can be found at: https://dhsprogram.com/data.

## Footnotes

1
https://www.g20.org

2
https://data.worldbank.org

## Appendix A

Table A1 Result of the CLMM fitted on complete cases only (*n* = 57,770).

**Table:**

*Factors*	Threshold 1: No Malnutrition|Moderate Malnutrition			Threshold 2: Moderate Malnutrition|Severe Malnutrition			Threshold 3: Severe Malnutrition|Not Alive		
	OR	95% CI	*p*-value	OR	95% CI	*p*-value	OR	95% CI	*p*-value
*Child’s sex at birth (b4)–Base category: male*									
Female	0.98	[0.94–1.01]	0.237	0.98	[0.94–1.01]	0.237	0.98	[0.94–1.01]	0.237
*Birth weight–Base category: Normal birth weight*									
Low birth weight	0.62	[0.59–0.65]	0.000	0.52	[0.49–0.56]	0.000	0.45	[0.42–0.49]	0.000
*Birth order–Base category: First*									
Second or third	1.24	[1.18–1.29]	0.000	1.11	[1.03–1.2]	0.005	1.10	[1.01–1.21]	0.031
Fourth or more	1.13	[1.05–1.22]	0.001	0.93	[0.83–1.03]	0.155	0.71	[0.62–0.81]	0.000
*Mother’s current age (baseline)–Base category: 15–24*									
25–34	0.83	[0.79–0.87]	0.000	0.94	[0.88–1.02]	0.127	1.42	[1.3–1.55]	0.000
35–49	0.70	[0.65–0.75]	0.000	0.69	[0.62–0.77]	0.000	1.27	[1.1–1.46]	0.001
*Highest educational level of mother (v106)–Base category: No education*									
Primary education	1.32	[1.23–1.42]	0.000	1.18	[1.07–1.3]	0.001	1.08	[0.96–1.22]	0.177
Secondary education	1.54	[1.45–1.64]	0.000	1.38	[1.28–1.5]	0.000	1.40	[1.27–1.55]	0.000
Higher education	2.00	[1.85–2.16]	0.000	1.38	[1.28–1.5]	0.000	1.57	[1.35–1.82]	0.000
*Full ANC status–Base category: Yes*									
No	1.02	[0.97–1.06]	0.482	0.95	[0.89–1.02]	0.150	0.88	[0.81–0.95]	0.002
*Place of delivery–Base category: Institutional*									
Non-Institutional	0.78	[0.71–0.85]	0.000	0.90	[0.81–1]	0.055	0.92	[0.8–1.05]	0.224
*Cooking fuel–Base category: Safe*									
Unsafe	0.83	[0.79–0.87]	0.000	0.93	[0.86–1]	0.065	0.88	[0.8–0.97]	0.007
*Type of place of residence (V025)–Base category: urban*									
Rural	0.78	[0.74–0.82]	0.000	0.90	[0.83–0.98]	0.012	0.99	[0.9–1.08]	0.765
*Wealth index combined (v190)–Base category: Poorest*									
Poorer	2.01	[1.89–2.13]	0.000	1.15	[1.06–1.25]	0.001	0.98	[0.89–1.09]	0.749
Middle	3.30	[3.08–3.54]	0.000	1.51	[1.37–1.67]	0.000	1.28	[1.13–1.44]	0.000
Richer	4.96	[4.59–5.35]	0.000	1.71	[1.53–1.91]	0.000	1.24	[1.08–1.42]	0.002
Richest	9.18	[8.36–10.07]	0.000	3.09	[2.67–3.57]	0.000	2.31	[1.93–2.77]	0.000
*Intercept*	0.69	[0.54–0.9]	0.005	10.53	[7.99–13.88]	0.000	19.66	[14.63–26.43]	0.000
*Estimate of random effect variance [95% CI]*							0.441 [0.260–0.799]		
*Model fit* AIC: 96760.18 BIC: 97236.88									

OR, Odds Ratio.
